# Palmitoylethanolamide in Human and Animal Obesity

**DOI:** 10.3390/molecules31142524

**Published:** 2026-07-20

**Authors:** Clara Naccari, Elettra Mancuso, Eugenio Donato Di Paola, Giovambattista De Sarro

**Affiliations:** Department of Health Sciences, University “Magna Græcia” of Catanzaro, 88100 Catanzaro, Italy; elettramancuso@unicz.it (E.M.); dipaola@unicz.it (E.D.D.P.); desarro@unicz.it (G.D.S.)

**Keywords:** obesity, human, animals, anti-obesity drugs, PEA

## Abstract

*Background:* With the increase in the middle-aged population and sedentary lifestyle, a high incidence of obesity has been observed in humans and in animals. Obesity is consequent or correlated to multiple diseases, such as metabolic-dysfunction-associated fatty liver disease (MASLD), diabetes, dyslipidemia, etc. The attention of many researchers is focused on understanding the specific cellular mechanism and the role of inflammation, particularly chronic, in the development of this pathology as well as its link with dysmetabolic conditions, which seriously affect the survival of both humans and animals. *Objective:* The aim of this review is to discuss the mechanism responsible for obesity, the specific drugs used in the treatment of this disease, and, considering the link between obesity and inflammation, the possible employment of Palmitoylethanolamide (PEA), a natural lipidic mediator with anti-obesity activity in humans and animals. *Materials and Methods:* The selection of articles chosen for this review paper was performed through the most important electronic databases (PubMed, Scopus, Web of Science, and Google Scholar); the specific inclusion criteria were applied systematically each time to ensure that the selection of papers closely aligned. *Results:* The treatment of obesity is focused on the management of weight through dietary caloric restriction, sustainable nutritional behaviors and long life therapy, which are also useful to prevent comorbidities. Several specific drugs for the treatment of this pathologic condition are available in both human and veterinary medicine. However, considering the documented link between inflammation and obesity, the possible use of PEA, authorized in veterinary medicine as a food supplement, could represent a valid therapeutic strategy in the treatment of human obesity. *Conclusions:* From studies present in the literature on obesity and its therapeutic approach in both human and veterinary medicine, and considering the importance of natural molecules in health management, the use of PEA as a dietary supplement, for its anorexic and fat-losing properties, could be considered a valid tool to counteract overweight and obesity in humans and animals and to avoid the onset of consequent comorbidities.

## 1. Introduction

Obesity is defined, according to the World Health Organization (WHO), as abnormal or excessive fat accumulation that represents a risk to health. It is a complex, multifactorial, progressive and chronic disease, whose incidence has significantly increased in the last century and has reached epidemic levels globally. In fact, it is considered one of the most important problems for public health. The evaluation of obesity is correlated to body mass index (BMI), ranging in humans from 25.0 to 29.9 in overweight and ≥30.0 in obesity, according to WHO guidelines. In general, obesity is classified as class I for BMI 30.0–34.9, class II for BMI 35.0–39.9 and class III for BMI ≥ 40.0 [1]. The presence of excessive fat deposition in the abdominal region, commonly known as “abdominal obesity”, is indicative of a greater risk for health, particularly cardiovascular disease [2]. Obesity can be considered a consequence of dysregulation between food intake and energy consumption, resulting in an excessive amount of white adipose tissue (WAT) [3,4,5], with consequent biochemical alterations, the release of inflammatory mediators directly involved in the pathophysiology of obesity, and the appearance of metabolic diseases responsible for comorbidities. In fact, obesity is followed by an increased risk of type 2 diabetes, heart and cardiovascular disease, endocrine dysfunction, hypertension, dysmetabolic symptomatology, cerebral vasculopathy, arthropathy, cancer, etc. Therefore, considering the general body status, obesity quickly leads to a negative impact on quality of life, sleeping, moving, respiratory activity, reproduction, etc. [6,7,8].

Today, obesity is considered a pathologic condition with multifactorial etiology, including genetic, food and environmental factors and also behavioral and psychological influence [9]. Particularly, the attention is focused also on socio-economic aspects, such as sedentary lifestyle and calorie-rich diets, which can predispose one to obesity [10,11]. For example, the globalization of food systems is one of the main topics because it is responsible for the production of more processed and affordable food; in addition, this new food culture promotes overconsumption, increased food waste and unequal distribution of nutritional resources among rich and poor countries [12]. This aspect, together with bad eating habits, such as preferring frozen, fat-rich and pre-cooked foods, contributes directly to obesity onset and spread.

However, another emerging aspect and an object of interest among researchers is that the specific socio-economic and behavioral factors listed above favor obesity in both humans and animals. Recently, this problem has also been of interest in veterinary medicine, in the cure and management of pet animals [13]; in fact, the Association for Pet Obesity Prevention documented in 2022 that 61% of cats and 59% of dogs in the U.S. are overweight or obese [14]. These species are considered important sentinels because their health status reflects that of the owners and their food and domestic habits [15]. For this reason, the prevention and treatment of obesity is addressed with a valid collaboration between human and veterinary medicine, according to a *One Health* approach [16].

This review aims to discuss the mechanism responsible for obesity, the incidence of this disease in humans and animals (pets, sport and farms), and the specific drugs available for the treatment of this disease. In detail, considering the link between obesity and inflammation, as documented in the literature, this review is focused on the possible use of Palmitoylethanolamide (PEA), a natural lipidic mediator with anti-obesity activity both *in vivo* and *in vitro*, and its ability to act on inflammation associated with obesity, as a promising therapeutic strategy to counteract overweight and obesity in humans and animals and to avoid the onset of consequent comorbidities.

## 2. Materials and Methods

The selection of articles chosen for this review paper was performed through electronic databases, including PubMed, Scopus, Web of Science, and Google Scholar. To identify the most relevant papers on the topic of this review, the keywords used were the following: “obesity”, “overweight”, “mechanism”, “inflammation”, “adipose tissue”, “incidence”, “epidemiology”, “therapy”, “anti-obesity drugs”, “natural anti-obesity molecules”, and “Palmitoylethanolamide”. In addition, a combination of specific and broad search terms was used, particularly “human obesity”, “animal obesity”, “human anti-obesity drugs”, “veterinary anti-obesity drugs”, and “Palmitoylethanolamide and anti-obesity effects”.

An initial preliminary selection of articles was conducted according to the title and abstract; subsequently, a more detailed search of articles was carried out, taking into account the specific topic of the sub-sections proposed in this review.

The inclusion criteria comprised peer-reviewed articles, reviews, and original research papers published in English and available in full text. Conversely, articles not written in English or those outdated were excluded. For each article, the title, abstract and full-text assessments were performed before inclusion in this study. These criteria were applied systematically throughout the process to ensure the selection of closely aligned papers.

The selected articles were descriptive of overweight and obesity in humans and animals, their incidence, actual pharmacological therapy in human and veterinary medicine, and possible innovative strategies involving natural products with anti-obesity properties, such as PEA.

Although the majority of the articles present in the literature were related to studies involving animal models, special attention was given to clinical trials in both human and veterinary medicine.

## 3. Mechanism of Obesity

**Figure 1:**
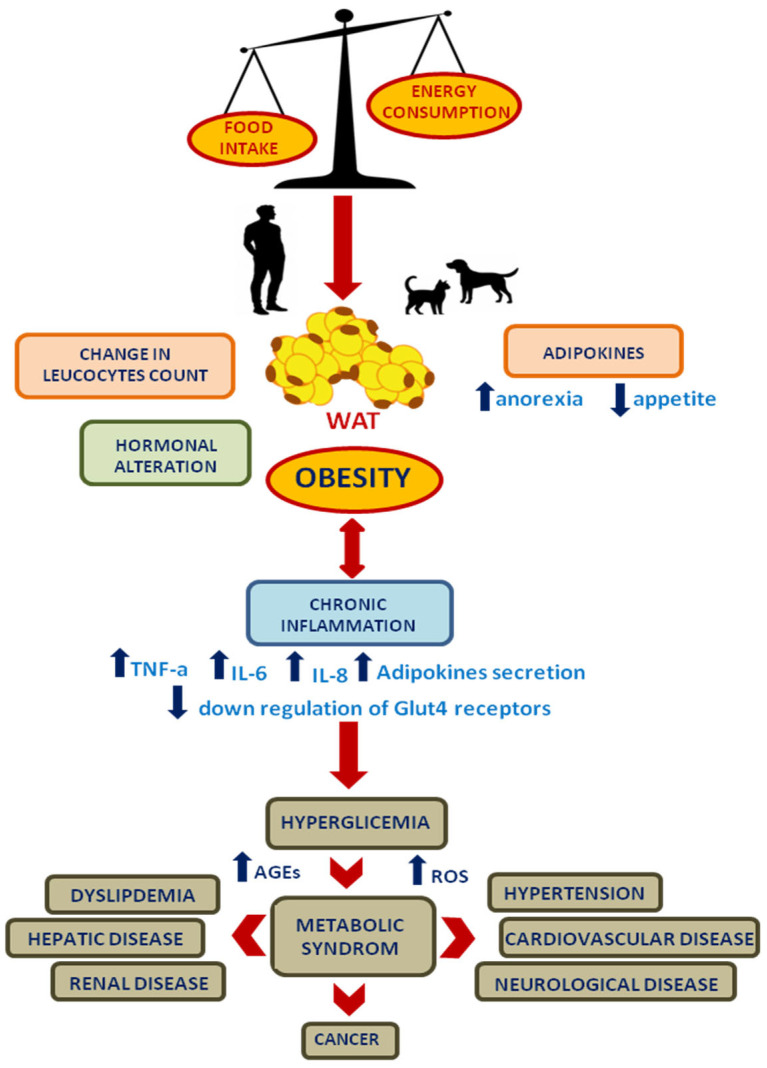
Schematic pathophysiological illustration of obesity onset and related comorbidities (WAT: white adipose tissue; TNF-α: tumor necrosis factor, IL-6: interleukin 6; IL-8: interleukin 8; AGEs: advanced glycated end-products; ROS: reactive oxygen species. The up/down arrow is indicative of increase/decrease production).

From a physiopathological point of view, obesity can be considered a consequence of a dysregulation between food intake and energy expenditure, resulting in an excessive amount of white adipose tissue [3,4,5]. The adipose tissue is constituted mainly of white adipose tissue (WAT), which is located in subcutaneous and perivisceral level, and brown adipose tissue (BAT), which is present in the cervical, thoracic, mediastinal, and abdominal areas, exerting opposite functions. Specifically, the first, WAT, is involved in energy storage and homeostasis in response to nutritional demands and acts as an endocrine organ by producing hormones and cytokines with regulatory functions, such as the appetite control and inflammatory responses [17]; conversely, the second, BAT, is responsible for regulating thermal insulation for adaptive thermogenesis, body temperature, and glucose and lipid metabolism, thereby playing a major anti-obesity and anti-diabetes role [18,19]. An increased production of WAT can alter adipokines production and, consequently, promote the secretion of pro-inflammatory cytokines. As WAT is an endocrine organ, the increased release of inflammatory mediators plays a key role in the patho-physiology of obesity, and is directly linked to the development of metabolic diseases. A characteristic condition observed in obesity is an evident chronic inflammation of adipose tissue coupled with nutritional excess, which results in alterations to the hormonal and immune systems, particularly changes in leukocyte count (monocytes, lymphocytes and neutrophils), reduced B- and T-cell proliferation, etc. [20]. This chronic inflammatory state is characterized by a great infiltration of macrophages into the adipose tissue, which contributes to the secretion of inflammatory cytokines (TNF-a, IL-6, IL-8, etc.). Several studies have demonstrated that immune dysfunction, which is linked to the appearance of inflammation and insulin resistance, is a condition associated with obesity. In detail, the fat accumulation in adipocytes causes oxidative stress, which modifies adipokines secretion and downregulates the Glut-4receptors, with the consequent appearance of insulin resistance and metabolic syndrome [21]. The condition of hyperglycemia may cause glycoxidation of different molecules and the formation of advanced glycated end-products (AGEs). These products exert pro-inflammatory and pro-oxidant effects, stimulating the secretion of inflammatory cytokines and activating the production of reactive oxygen species (ROS) [22,23]. Consequently, the excessive generation of ROS through various mechanisms (NADPH oxidases, oxidative phosphorylation, protein kinase-C activation, glyceraldehydes auto-oxidation pathways, etc.) is responsible for subsequent cellular oxidative stress and pro-inflammatory status [24,25]. In addition, the inflammatory, endocrine, and metabolic disorders consequent to obesity can enhance the risks of cancer [26] (Figure 1).

The link between obesity and cancer is not well known; however, this pathological condition determines DNA damage and/or alteration of its repair pathways. In fact, the increased production of ROS in obese subjects, such as superoxide (O_2_•−), hydroxyl radical (HO•), and lipid(hydro)peroxides(LOOH), can participate in lipid peroxidation. This results in the consequent production of etheno-, propano- and malondialdehyde, which are able to interact with DNA, forming oxidized bases, DNA-adducts and causing subsequent damages [27,28,29]. The ability of radical species to induce tissue oxidative stress, with alterations to the structure of carbohydrates, proteins, phospholipids and nucleic acids, as well as mitochondrial oxidation, genomic damage and mutations, could be the basis of cancer onset [30,31]. Generally, it is common to speak about “obesity-related cancers”, considering that the obesity pathology involves increased serum levels of glucose, insulin, lipids, leptin and inflammatory cytokines, which are able to promote cancer pathogenesis.

In this context, diet and eating habits play a key role; among these, the Mediterranean diet provides nutrients with protective effect that are able to support adequate DNA repair. In addition, obesity is often associated with aging [32], a condition characterized by an increased production of pro-inflammatory molecules, responsible for the adipose tissue increase. It has been documented that obesity, by altering the metabolic balance, promotes cellular processes involved in aging [33]. Therefore, the acceleration of cellular processes typical of aging, together with inflammation and oxidative stress, can significantly contribute to dysregulation of DNA repair pathways, resulting in metabolic disorders and cancer development [34,35,36]. As demonstrated by epidemiological studies, several pathological conditions can promote obesity, being directly correlated to an increase in body index, such as metabolic dysfunction-associated steatotic liver disease (MASLD), diabetes mellitus, cardiovascular, musculoskeletal and kidney disease, etc. [37,38,39]. Furthermore, physiologic conditions, such as maternal nutritional imbalances during gestation and lactation are able to promote obesity, contributing to sex-specific metabolic alterations [40]. As previously reported, obesity is considered a multifactorial pathologic condition (involving genetic, dietary, environmental, socio-economic, and psychological factors) [41]; however, specific socio-economic aspects (sedentary lifestyle, calorie-rich diet, etc.) [9,10,11] and bad eating habits (such as the consumption of frozen and/or pre-cooked foods) contribute to the onset of obesity through similar mechanisms in both humans and animals.

## 4. Incidence of Obesity in Human

Since the second half of the last century, due to the development of the food industry and the concomitant consumption of processed food rich in calories and poor in nutrients, obesity and its consequent comorbidities are becoming common among the Western population [42,43]. The prevalence of obesity has doubled since the 1980s; currently, roughly one-third of the global population is classified as overweight or obese. A WHO report 2022; confirmed that approximately 1 billion people were obese, exactly 650 million adults, 340 million adolescents and 39 million children [44] and these percentages have likely continued to rise in recent years. Considering the 1990–2022 period, the prevalence of obesity has more than doubled: from 18.6% in 1990 to 42.0% in 2022 in the United States; from 12.7% in 1990 to 30.2% in 2022 in Australia; from 6% in 1990 to 28.1% in 2022 in Brazil; from1.5% in 1990 to 7.3% in 2022 in the Republic of Korea [45].

According to a WHO report on obesity in Europe published in May 2022 [46], a higher prevalence was observed in women, and the rate of obese subjects increased with age, reaching the maximum between 50 to 65 years. A previous epidemiological study on obesity (from 1980 to 2015), which utilized data from the Global Burden of Disease Study (Institute for Health Metrics and Evaluation, Seattle, WA, USA) [47], evaluated the increase in obesity according to the age, sex, several geographical areas (Africa, Americas, Eastern Mediterranean, European, Southeast Asia and Western Pacific), ethnicity, and socio-economic status. This study showed a higher prevalence of overweight women among both young and adult subjects (aged 20 and 44 years), confirming previous data [48]. However, this trend changed after the age of 45–49 years, with a prevalence in women, probably due to hormonal changes correlated to age, affecting the distribution and mobilization of adipose tissue storage, different insulin sensitivity, altered lipoprotein profiles, etc. [49]. In addition, the prevalence of obesity was greater among older individuals and women but not significantly correlated to geographical areas.

Considering, instead, the differences in lifestyle, dietary habits and socio-economic status between urban and rural populations or rich and poor countries (such as accessibility to fast-food restaurants and/or supermarkets, food education, economic disparity, etc.), no significant differences were observed compared to age and sex.

Relating to the worldwide incidence of obesity, in recent years it has also spread in poor regions due to the economic growth and increased urbanization in these areas. In a study by Yu Chung Chooi et al. [47], the obesity rate in the U.S. and U.K. populations from 1980 to 2015 remained at approximately 30–34% and 23–24%, respectively. In contrast, an increase was observed in Asian and Middle Eastern regions, such as Turkey (28.5%) South Africa (30.8%), Iraq (31.9%), U.S. (33.6%), and Egypt (35.3%) as well as in Latin America, including Mexico (28.6%), Brazil (22.6%) and Argentina (23.2%). In a study by Boutari and Mantzoros [48], epidemiological data from 1980 to 2019 showed an increase in obesity from 6.8% to 22.4% in the Americas, from 6.4% to 17.4% in the Eastern Mediterranean region, and from 3.8% to 10.9% to 2019 in the African region. Recently, a rapid and substantial increase in obesity was also documented in the Chinese population, although the rate remains lower than that in the USA [50].

However, a general increase in obesity among 2019 and 2022 worldwide could be correlated to the COVID-19 pandemic. This trend is attributed to the behavioral changes in the population (such as lack of physical exercise, increased home cooking, higher food consumption, prolonged time spent indoors, sedentary habits, etc.) [51], which increases the risk of severe disease, hospitalization, and death [52,53,54].

Although the different increases in obesity in specific regions of the word area consequence of several factors previously described, the widespread diffusion of obesity, considered a public health concern [55], needs adequate prevention strategies and food education campaigns.

## 5. Emergent Obesity Condition in Animals

Obesity is becoming an emerging problem also in veterinary medicine. In pets, the prevalence of obesity has increased worldwide in the last decade [56] and it is currently considered the most common nutritional disorder in animals [57]. The Association for Pet Obesity Prevention documented an increase in this condition; in fact, in 2022, 61% of cats and 59% of dogs in the U.S. were classified as overweight or obese [14]. The onset of this condition in pets is mainly attributable to diet and lifestyle habits, particularly daily food intake, nutrient absorption and maintenance of the energetic homeostasis [58,59]. Other important factors include genetics, sex, fat distribution, and the use of drugs (e.g., drug-induced polyphagia caused by glucocorticoids and anticonvulsants). In addition, the prevalence of obesity increases with animal age and as consequence of surgical treatments [60]; furthermore, neutering is also considered a significant risk factor [57].

This condition was further influenced by pandemic-related inactivity and overfeeding during COVID-19 [61], as well as a consequence of underestimated body condition. In fact, prior to the pandemic, a study carried out on the canine population in France reported a 38.8% prevalence of overweight dogs [62]; however, a general increase in pet obesity is currently reported worldwide [63,64].

Similar to humans, obesity, which is highly prevalent in older companion animals, is correlated with the development of several other comorbidities. These include cardiac diseases, hypertension, dyslipidemia and metabolic disorders (such as hypothyroidism, hyperadrenocorticism, etc.), which severely affect animal survival and longevity, with consequent emotional impact of owners and significant expenses for pharmacological treatment [60]. In dogs, the main risks of comorbidities include diabetes mellitus, calcium oxalate-urolithiasis, cardiac damages, orthopedic alterations and neoplasia (predominantly mammary tumors) [56,65,66,67,68]; in cats, instead, the main risks are arthritis, musculoskeletal alterations, cardiac diseases, urinary damage and allergic conditions (feline asthma) [69,70,71,72]. Recently, the emergence of cognitive and behavioral syndromes was documented in obese animals, including, particularly, depression [73].

For the diagnosis of pet obesity, clinic veterinarians use the Body Condition Score (BCS), a semi-quantitative method employed to evaluate an animal’s body composition, percentage of body fat and muscle, via palpation, anatomic landmarks, and overall shape. To assess overweight status, two recognized BCS scales are employed, according to the following scoring: 5-point scale (1: very thin; 2: underweight; 3: idealweight; 4: overweight, 5: obese) and 9-point scale (1–3: too thin; 4–6: ideal weight; 7–9: overweight) [74,75,76,77].

Although the attention is focused on the prevalence of obesity in companion animals, based on the owner’s information, this condition is also of great interest in sport animals, particularly equines. In fact, obesity is considered one of the most prevalent health and welfare issues affecting horses and ponies, but the true incidence of this pathology is currently underestimated [78]. Studies conducted in the U.K. have documented that 31 to 54% of equines [79] and 72% of adult ponies are overweight or obese [80]; similar data were obtained from the equine populations of other northern European countries [81,82]. The onset of this condition is correlated to key factors, such as inadequate exercise, great intake of highly digestible food, management, etc. In companion and performance horses and ponies, overweight or obesity constitute a high risk factor for the subsequent development of equine metabolic syndrome, insulin dysregulation and laminitis [83,84]. At the same time, this pathologic condition is also responsible for negative impact on the athletic performance in sport animals because the excessive adipose tissue increases workload effort and compromises limb health [85]. The adiposity evaluation in horses is carried out through morphometric measurements, ultrasonography of subcutaneous fat, bioelectric impedance, etc. [86]. The most common methods used to determine equine fat coverage are represented by BCS and Cresty Neck Scores (CNS). For equine BCS, two scoring systems exist: the 9-point, known as *Henneke system* (1–3: very poor; 4–5: being ideal; 6–7: overweight; 8–9: obese) [87], and the 6-point or *Carroll & Huntingdon system* [88] (1–2: very poor; 3–4: good; 5–6: very fat). Instead, the CNS is a 0–5 scale used to measure regional adiposity via nuchal fat accumulation (0: no visible or palpable; 1: no visible crest but slight filling on palpation; 2: visible crest with fat evenly distributed; 3: thick crest and fat deposited in the center of the neck; 4: very thick crest; 5: massive and fat-filled crest drooping to one side) [86].

Finally, obesity is also a critical issue in farm animals. It is due to the overfeeding intended to maximize the production of animal-derived foods, with consequent metabolic alterations and fertility problems in livestock [89], negatively impacting zootechnical production. Overfeeding livestock is a common practice in intensive farming employed to accelerate animal growth and increase production of animal-derived foods, to satisfy increased market demand. However, the issue of overweight and obese farm animals is not only correlated to compromised animal health and welfare but it is also responsible for serious environmental damage, due to an increased food waste production and CO_2_ release [90]. Furthermore, it exerts an important impact on consumers of animal-derived foods. As a consequence of modified breeding and feeding practices, these food products are often less appreciated by consumers [91] and their assumption could represent a risk for human health, due to their low nutritional value. For example, the overnutrition of farm animals with diets rich in cereals but poor in forage could result in foods with a lower content of healthy fatty acids, such as omega-3, and higher levels of saturated fats. Overfeeding farm animals can cause metabolic imbalances and alteration of immune systems, making them more susceptible to disease, with an increased risk of infections, antimicrobial resistance and zoonosis [92,93]. Furthermore, due to the altered nutritional balance, the animals could require nutritional supplements and also pharmaceuticals, leading to the possible presence of residual drug levels in their meat and animal-derived food.

To control obesity in veterinary medicine, the issue must be holistically analyzed through a multidisciplinary approach, comparative studies, informative campaigns, etc. Considering that there are no interventions capable of preventing or combating this complex multifactorial problem, it has been hypothesized to manage obesity according to a “*One health*” approach. Comparative clinical research on obesity and its comorbidities aims to obtain useful information for both companion animals and their human owners [70], according to the guidelines of the World Small Animal Veterinary Association-One Health Committee.

The research carried out on pet obesity is numerically limited and less robust compared to human studies, but is useful for understanding key factors involved in development of this pathologic condition. Obesity in pets may also be influenced by cultural factors and could be considered as an extension of the human–animal relationship [94]. A study by Bartges et al. [95] describes this interdisciplinary “*One health*” model in detail to promote the health of people, animals and environment. By using comparative and translational clinical research, this model aims to realize the long-term weight loss maintenance and adherence to weight loss regimes through a strategy called “*The People and Pets Exercising Together*”. Another study by Arena et al. [96] assessed and predicted potential factors in the development of feline overweight status, such as occurrence of urinary tract diseases, arthritis, dermatopathy, diabetes mellitus, hepatic lipidosis, neoplasia, gastrointestinal, cardiac, musculoskeletal and oral diseases. Additionally, it examined living and environment features (such as the presence of other animals), behavioral factors (e.g., anxiety and depression), as coadjuvant in obesity development, and correlating owner management with cat behavior and welfare. Through this multidisciplinary approach, it is possible to provide useful information to veterinarians [97] and owners to better monitor animal weight, feeding, and lifestyle, to guide the correct choice in animal feed, underling the importance of quality and safety of products to their diet.

Finally, the researchers’ interest in animal obesity is linked to the health and welfare of human owners. Currently, various risk factors for overweight and obesity are recognized and classified into five categories: pet-related, owner-related, diet-related, environment-related, and activity-related. The first, pet-related risk factors, includes a correlation among canine and feline obesity and age, sex and breed; the second, owner-risk factors, associates pet obesity with owner’s age, level of education or specific habits (for example, allowing the dog to sleep in owners’ beds). The third, diet-related risk factors, is common to animals and owners and includes the consumption of low-cost food, frequent treats, etc. The fourth, environmental risk factors, could involve the presence of multiple individuals and/or other animals in the household, while the fifth, the activity-related risk factors, includes the low physical activity levels and/or the inability to exercise freely [62]. In this context, the cultural influences are very important for both pet owners and their animals. For instance, Suarez et al. [94] conducted a comparative study on overweight and obesity in the population of Thailand and the Netherlands and observed that in the first, pets owners are less engaged in outdoor activities with their dogs due to the high temperatures with respect to colder countries, with consequent negative impact also on the dog’s quality of life.

## 6. Drugs Used in Human Obesity

Obesity is strongly correlated with the development of serious and often lethal pathologies (including cardiovascular diseases, type 2 diabetes, dyslipidemic syndrome, asthma and cancer) and requires specific lifelong therapy for weight management. According to the Obesity and Endocrinology Societies, anti-obesity medications (AOMs) are recommended for individuals with a BMI ≥ 30 kg/m^2^ or if ≥27 kg/m^2^ in the presence of one or more comorbidities. The first therapeutic step is an initial management of overweight and obesity, by adopting sustainable nutritional behaviors, regular physical activity and healthy lifestyle habits to reduce body weight and cardio metabolic risk [98]. Particularly, patients are submitted to a dietary caloric restriction for the purpose of reducing weight, enhancing adipose tissue plasticity and normalizing endocrine function. In some cases, bariatric surgery is useful for reducing weight in patients with a BMI > 40 kg/m^2^ alone or >35 kg/m^2^ with comorbidities, or in those not responsive to dietary caloric restriction protocol [99,100]. This procedure is often preferred to gastric banding and sleeve gastrectomy because these last can cause malabsorption. The use of specificdrugs appears to be a crucial tool to enhance weight loss in short- and long-term treatment. Currently, the specific lifelong pharmacological treatment aims to affect pathophysiological pathways that lead to obesity, to regulate weight and to prevent comorbidities.

From 1950 to the present, several AOMs were approved by the FDA: for short-term treatment the molecules used are Phentermine and Cathin hydrochloride, while for long-term: Sibutramine, Fenfluramine, Rimonabant and Orlistat [101]. Subsequently, several drug classes have been investigated for obesity treatment, including mitochondrial uncouplers, sympathomimetics, serotonergic agonists, lipase inhibitors and cannabinoid receptor antagonists [102]. Generally, these agents are grouped according to three main mechanisms of action: appetite suppression, fat absorption reduction, and fullness and satiety increase (Table 1).

Among drugs acting on appetite suppression, Phentermine and Diethylpropionate sympathomimetic drugs used for short-term weight loss due to their possible side effects. A comparative study on their effectiveness in the treatment of obese patients treated once daily with either 30 mg orally of Phentermine (group 1) or a 75 mg tablet of Diethylpropion (group 2) over a 12-week period demonstrated greater weight loss in patients treated with Phentermine [103]. This drug has also been used in association with Topiramate, a carbonic anhydrase inhibitor. Originally used for epilepsy and migraine prevention, Topiramate showed efficacy and safety in promoting weight loss for overweight and obesity management. In a study on adolescents with severe obesity treated with Topiramate (75–100 mg daily), a clinically significant BMI reduction of approximately 4–6% was observed over 6 months, with acceptable tolerability. The first combination of Phentermine–Topiramate for obesity was approved by the FDA in 2012 for long-term management [104,115], to employ with a reduced–calorie diet and increased physical activity in patients with an initial BMI over 30 kg/m^2^ or over 27 kg/m^2^ in the presence of comorbidity. This combination mainly suppresses appetite, although its specific mechanism of action in human is not well known; however, animal studies indicate that this association acts as an appetite suppressant by enhancing the release of biogenicamines (norepinephrine, dopamine and serotonin) in the CNS [116,117]. Phentermine has been recently withdrawn in the U.K. and France, and EMA revoked its marketing authorization in the EU; however, it is still used in the U.S., Australia, South Korea, and Mexico. Bupropion–Naltrexone is another association approved for chronic weight management in adults with obesity or overweight and weight-related comorbidities [118]. Bupropion is an antidepressant able to decrease food intake with an anorectic mechanism due to the inhibition of dopamine and reuptake of norepinephrine [105]. Naltrexone, instead, is an opioid receptors antagonist, with an appetite suppressant action mediated by β-endorphin. In a study conducted on healthy overweight women (BMI ≤ 27–40 kg/m^2^), the treatment with daily doses of combined Bupropion (360 mg) and Naltrexone (32 mg) in a trilayer tablet improved control over eating behaviors [106]. Specifically, these drugs in combination act synergically on two distinct brain areas, the hunger center and the reward system, to reduce appetite, increase self-control and enhance satiety signals. Clinical studies carried out on patients with a BMI ≥ 27 kg/m^2^ and at least one weight-related comorbidity, such as hypertension, documented a significant percentage of weight loss after assumption of Bupropion–Naltrexone (360/32 mg) for 56 weeks, compared to placebo [118,119,120].

Setmelanotide, a melanocortin-4-receptor agonist, acts on several areas of hypothalamus, specifically in the paraventricular nucleus and lateral hypothalamic area, decreasing appetite and regulating energy expenditure. Approved by the FDA in 2020 as a subcutaneous injectable formulation for chronic weight management, in obese patients of 6 years or older with leptin receptor (LEPR) deficiency, this drug is safe; in fact, its most common effects are injection-site reactions and skin hyperpigmentation. Several clinical trials have documented the effectiveness of this drug. For instance, a study by Clément et al. [107] on patients with obesity secondary to LEPR deficiency treated for 12 weeks documented a 43.7% reduction in hunger and a total body weight decrease (≥10%) in 45% of patients within one year. Instead, in a study on patients with Bardet Biedl or AlstromSyndrome and obesity defined by a BMI ≥ 30 kg/m^2^ (≥16 years) or bodyweigh t > 97th percentile for age and sex (for individuals aged 6–15years), treated subcutaneously once daily with Setmelanotide at age-adjusted doses (for individuals ≥ 16 years: firstly 2mg which increases to 3 mg at the beginning of week 3; for individuals < 16 years: 1 mg for the first week, which increases to 2 mg for the second week and to 3 mg at the beginning of the third week), a body weight decrease corresponding to 7.6% was observed within 1 year of treatment [121]. Setmelanotide represents the first drug approved by the FDA for the treatment of obesity of genetic origin [122].

Another drug approved by FDA in 2014 is Metreleptin, a leptin analogue, employed in patients with deficiency of this adipokine, with important metabolic effect and influences on immune, neuroendocrine and neurocognitive functions. The advantage of this drug is the possibility of a single daily subcutaneous injection. In a study by Grover et al. [108] on patients affected by lipodystrophy and low leptin levels treated with Metreleptin (5 mg subcutaneous injection every 12 h, for the next 14 days), this drug improved the metabolic parameters (including cholesterol, triglycerides, fasting glucose, insulin sensitivity) at 6 months from the treatment, although this trial was carried out on a limited number of participants.

Drugs acting on the reduction in fat absorption are Orlistat and Cetlistat, inhibitors of lipase, the enzyme responsible for breaking down triglycerides in the intestine. These drugs act in the lumen of the stomach and small intestine, preventing significant fat intestinal absorption and reducing calorie intake without affecting the appetite, and improving insulin sensitivity, lipid profile, and blood pressure. These AOMs have demonstrated efficacy and safety in several clinical studies, with the advantage of not causing adverse effects typical of anorectic drugs acting on the CNS (amphetamines). In a study by Jain et al. [109], the reduction in body weight observed in patients treated with Orlistat (120 mg three times a day, 1h before breakfast, lunch, and dinner) was 5.63% compared to placebo group (2.3%), and the decrease in cholesterol concentration was 10.68 mg/dL. Furthermore, the effectiveness of Orlistat has been documented in adolescents and adults with metabolic syndrome, pre-diabetics, type 2 diabetes and obesity. A double-blind, randomized, placebo-controlled, parallel-group study in healthy male volunteers with a BMI of 30 kg/m^2^, treated with different doses of Cetilistat (ranging from 50 to 240 mg for 5 days) alongside a controlled calorie diet, showed a reduction in dietary fat adsorption, and the drug was well tolerated at all doses [110]. In fact, although Orlistat and Cetlistat exhibit similar efficacy, the newer agent among them, Cetilistat, is better tolerated with a low incidence of gastrointestinal side effects compared to the other.

Drugs acting on the enhancement of fullness and satiety are mainly Liraglutide and Semaglutide, glucagon-like peptide receptor (GLP-1) agonists, which are able to lower the blood glucose levels to increase satiety and decrease appetite, delaying gastric emptying and reducing body weight in a dose-dependent manner [111]. These drugs were initially administrated for subcutaneous injection to treat type 2 diabetes [112] but, most recently, also to counteract obesity due to their ability to promote weight loss, regulate appetite, increase fullness and satiety, and stimulate energy expenditure [103].

Generally considered the most effective, Semaglutide has been approved as a once-weekly subcutaneous somministration (2.4 mg) for weight loss and glycemic control in individuals with obesity and related complications. Its primary observable adverse effects are nausea, diarrhea, vomiting and constipation. However, a randomized, controlled, phase 3 trial documented that administration of Semaglutide at a higher dose (7.2 mg) in individuals with obesity showed a significant body weight reduction, maintaining a favorable risk–benefit profile [123]. Instead, in a randomized, placebo-controlled, crossover study in diabetic individuals treated with Liraglutide, administered over 17 days at gradually increased doses (0.6 mg for 7 days,1.2 mg for 7 days, and 1.8 mg for 3 days), this protocol was able to minimize adverse effects (e.g., nausea), showing a central mechanism involved in metabolism regulation and weight loss [112].

Another drug, Tirzepatide, a GLP-1 and glucose-stimulated insulin-tropic peptide receptor agonist, was approved by the FDA for the treatment of type 2 diabetes as a once-weekly subcutaneous injectable peptide. It is employed in the treatment of obesity, stimulating pancreatic insulin release, reducing hyperglycemia and increasing adiponectin levels [113,124]. In a clinical trial, volunteers with overweight and a BMI of 30 kg/m^2^ or higher treated with Tirzepatide (5, 10 or 15 mg) subcutaneously once weekly for 72 weeks showed a reduction in body weight, alongside improvements in cardiovascular and metabolic risk factors (such as waist circumference, systolic and diastolic blood pressure, fasting insulin, lipid, aspartate-aminotransferase levels, etc.) [125].

Recently, the attention has been focused on Gelesis, a formulation of hydrogel particles designed to mimic the volumetric effects of raw vegetables, able to absorb water through a cellulose–citric acid matrix, to increase fullness; it is indicated for the management of overweight and obesity. In fact, in a clinical trial in adult volunteers with overweight or obesity treated orally with 2.25 g of Gelesis 20–30 min before lunch for 24 weeks [114], this formulation was able to expand the stomach and small intestine during the meal, promoting the sense of fullness and reducing the appetite, also showing a good safety and tolerability profile.

Most recently, the pharmacological treatments for obesity are categorized for monogenic syndromes, a severe condition due to mutations in genes implicated in the leptin–melanocortin pathway, and nonsyndromic obesity, a more rare and severe form, caused by mutations in single genes acting on the hypothalamus or the leptin–melanocortin pathway, thereby altering the energy homeostasis and causing endocrine dysfunction [115]. Specifically, drugs used in the monogenic obesity syndromes are Setmelanotide and Metreleptin.Instead, in nonsyndromic obesity, the AOMs commonly employed are Orlistat, Phentermine–Topiramate, Bupropion–Naltrexone, Liraglutide and Semaglutide.

## 7. Pharmacological Approach in Animal Obesity

The pharmacological approach to obesity in veterinary medicine has achieved limited success in pets due to the lack of owners’ compliance with therapy. Dietary modifications, increased exercise and changes in animal lifestyle [60] are traditionally considered the main tools in pet obesity treatment [126], although their effects are not visible in a short time. In general, animal obesity is not well recognized by owners, and the main therapeutic interest is the prevention of type 2 diabetes and other comorbidities associated with overweight (such as osteoarthritis, respiratory problems, cardiovascular disease, neoplasia, hepatic disease, etc.), to guarantee pets’ longevity. Another limit in the treatment is represented by the few available veterinary drugs compared to human medicine.

A drug authorized by the EMEA in 2006 for canine obesity is Mitratapide, belonging to the class of microsomal triglyceride transfer protein (MTP) inhibitors, able to reduce fat absorption. In a study conducted on beagle dogs, treated with oral somministration of Mitratapide (5 mg/mL once daily two times for 3 weeks, with an intermediate 14 days treatment-free interval), a significant weight reduction was observed through loss of adipose tissue, with a reverse effect on insulin resistance in dogs, but without effectiveness in appetite reducing [127]. In 2007, the FDA approved for the treatment of obesity in dogs the Dirlotapide, another MTP inhibitor, able to reduce appetite and decrease fat absorption [128,129,130]. This drug, administered orally for 4 weeks initially at 0.1 mg/kg (adjusted monthly during 24-week weight loss) in overweight Labrador retrievers, was safe and effective in the reduction and management of bodyweight [131].

An innovative approach in the treatment of pet obesity is represented by GLP-1 receptor agonists, drugs successfully used in human therapy. Due to similar metabolic pathways, GLP-1 receptor agonists are actually under investigation for their possible application in the management of animal obesity, particularly in cats and dogs. For the similar pathophysiology of human and feline diabetes, the attention is focused on Semaglutide, along-lasting GLP-1agonist, Tirzepatide, a GLP-1 agonist and glucose-dependent insulin tropic polypeptide (GIP) agonist, and Liraglutide, a GLP-1 receptor agonist. Specifically, Liraglutide is considered novel and effective for overweight canines, as documented in animal models to evaluate its weight loss effects. In a study carried out by Dik et al. [132] in obese dogs, the subcutaneous treatment with Liraglutide at a dose of 1.2 mg administered once daily for 40 days showed a significant reduction in BCS, body weight, appetite and cholesterol and triglyceride levels. Another molecule under investigation is Exenatide, a synthetic analog of exendin-4, which is a natural ligand of the GLP-1 receptor; in a study involving cats, this drug, injected subcutaneously (1 μg/kg), was able to improve glycemic control by reducing HbA1c levels, enhancing insulin secretion and lowering glucagon levels [133]. Considering the substantial efficacy of pharmacological treatment with GLP-1 receptor agonists in human medicine, these drugs should become important also in animal obesity, although the main approach in the management of pet obesity remains weight maintenance, dietary modifications and physical exercise. In this context, the use of nutritional supplements, such as Chromium Picolinate, could be useful together with a balanced diet. In fact, in an experimental study on normal-weight cats, Chromium Picolinate (150 ppb, 300 ppb, or 600 ppb) supplemented in the diet showed its ability to reduce glucose concentrations and improve glucose tolerance, suggesting its potential role in the management of feline obesity [134]. Also, Glycosaminoglycan dermatan sulfate could be considered a valid dietary supplement for its ability to attenuate diet-induced obesity [135,136]. In fact, studies on mice have shown that this complex carbohydrate, when administered as nutraceutical in the diet (30 mg/kg/day orally), counteracts the development of HFD-induced obesity and insulin resistance in obesity-prone mice by promoting energy expenditure [136].

## 8. Relation Between Obesity and Inflammation

**Figure 2:**
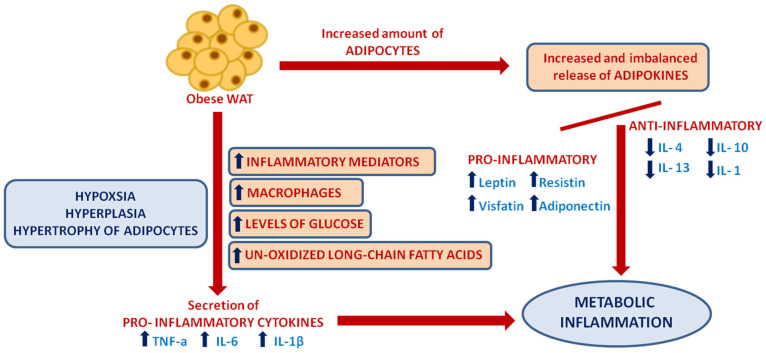
Schematic representation of the link between obesity and inflammation.

Inflammation plays an important role in obesity, contributing to the cellular and humoral response of adipocytes [137,138], and it is considered the link between obesity and metabolic syndrome [139,140]. As a consequence of the imbalance between excessive caloric intake and energy expenditure, there is an increased release of adipocytes [141]. As previously described, adipose tissue is mainly constituted of WAT, located in the subcutaneous and perivisceral levels, while the BAT is present in the cervical, thoracal, mediastinal, and abdominal areas [138,142,143]. In obesity, the excessive accumulation of WAT (which is actively involved in regulating physiologic and pathologic processes) causes an increased release of inflammatory mediators [3,144,145] and recruits macrophages, components of adipose tissue that regulate chronic inflammation and secretion of pro- and anti-inflammatory cytokines. The first evidence on the inflammatory origin of obesity is attributable to human and animal studies in the early 1990s, which demonstrated that the adipose tissue from both obese rodents and humans was characterized by inflammation and an increased secretion of pro-inflammatory cytokines, particularly TNF-α [146,147,148]. In response to an increased intake of nutrients, the adipose tissue exhibits hyperplasia, hypertrophy of adipocytes, hypoxia and altered balance between pro- and anti-inflammatory processes, resulting in the overproduction of pro-inflammatory mediators by macrophages [149], particularly TNF-α, IL-6, IL-1β, and acute-phase reactants, such as C-reactive protein (CRP) [139]. Among cytokines, TNF-α, leptin, resistin (or adipokine), visfatin and IL-6 are involved in promoting inflammation and are called “pro-inflammatory adipokines”, whereas transforming growth factor-beta (TGF-β), IL-4, IL-10, IL-13, IL-1 receptor antagonist (IL-1Ra) and adiponectin are considered “anti-inflammatory adipokines” [150,151]. Some studies on human have demonstrated the important role of IL-6 in the development of metabolic diseases and its link to obesity [152]. During acute-phase inflammation, this cytokine stimulates the hepatic production of CRP and the release of fibrinogen, white blood cells and platelets. CRP is considered a marker of systemic inflammation; therefore, high levels are correlated with the risk of metabolic diseases, myocardial damage and atherosclerotic events [153]. Adiponectin plays an important autocrine role: in the presence of high levels of IL-6, its release is reduced but it is negatively associated with obesity of rats (in both males and females), improving glucose metabolism and reducing inflammation, insulin resistance, uptake of nonesterified fatty acids and gluconeogenesis [154]. Generally, the increased release and circulation of adipokines and pro-inflammatory cytokines plays a key role in the onset of obesity and could be considered the true link between inflammation and obesity [155]. At the same time, the high nutritional rate and body weight lead to increased levels of glucose and unoxidized long-chain fatty acids, which contribute to stimulate the synthesis of pro-inflammatory adipokines [156], secreted by adipocytes through the modulation of inflammatory response (Figure 2).

The inflammation induced by obesity is specifically known as “obesity-induced low-grade chronic inflammation of the adipose tissue”, also called “meta-inflammation” (meaning metabolically-triggered inflammation), and is characterized by fibrosis, hypoxia, altered angiogenesis, etc. [138,157]. However, today, the detailed mechanism of chronic inflammation of adipose tissue linked to obesity remains poorly understood. In a clinical study by Petrus et al. [158] conducted on obese and nonobese women, glutamine was found to be downregulated in obesity and inversely associated with the WAT phenotype; furthermore, its *in vitro* and *in vivo* administration reduced pro-inflammatory gene and protein levels in adipocytes as well as macrophage infiltration. Therefore, the reduced glutamine levels in adipose tissue of obese women increase nuclear O-GlcNAcylation in adipocytes and the transcription of pro-inflammatory molecules.

The immune system is also involved in obesity [159]; in fact, the adipose tissue contains several immune cells that maintain the integrity and hormonal sensitivity of adipocytes and the activity of T-lymphocytes. These cells control the cytokine cascades that regulate eosinophils, mast cells, and macrophages in a polarized or activated state. In obese mice, several changes have been observed in the activation state and number of macrophages, accompanied by an increased production of cytokines such as TNF-α, indicative of adipose tissue inflammation and associated with insulin resistance and metabolic disease [160].

The chronic low grade of inflammation typical of obesity is at the origin of atherosclerosis, coronary artery disease, diabetes, etc [155]. These pathological conditions are interrelated because the adipose tissue is a endocrine organ involved in regulation of appetite, energy expenditure, reproductive and endocrine functions, inflammation, immunity and visceral fat expansion, with this last responsible for systemic inflammation, enhancing the production of free fatty acids [20].

The meta-inflammation typical of obesity is correlated with several dietary factors, including saturated fatty acids, sugars and altered gut microbiota profiles, all characteristic of dysfunctional immunity [161]. To explain the link between meta-inflammation and obesity, the attention has been focused on Nod-Like Receptor Protein 3 inflammasome (NLRP3), considered a sensor of metabolic danger signals [162], because recent data indicated NLRP3-dependent inflammatory pathways in meta-inflammation [163]. NLRP-3 is an intracellular inflammasome, a protein complex present also in adipose tissue, involved in the release of pro-inflammatory interleukins (IL-1β and IL-18) and innate immune response. It is active as a consequence of numerous signals in inflammatory and autoimmune diseases, metabolic disorders (such as metabolic syndrome, obesity, diabetes and gout), cardiovascular and also neurodegenerative diseases (including Parkinson’s disease, Alzheimer’s disease, etc.) [164,165]. NLRP3 possesses a pyrin domain 3 expressed in the cytosol of innate immune cells but is activated by the production of ROS. In presence of ROS, it stimulates the inflammatory protease, caspase-1, and the pro-inflammatory cytokines, IL-1ß and IL-18, which can contribute to development of inflammatory disease, such as rheumatoid arthritis, inflammatory bowel disease (IBD), multiple sclerosis, etc. [166]. All macronutrients introduced with the diet, carbohydrates, lipids and also cellular metabolites, can regulate NLRP3 inflammasome. In fact, the excess nutrients alter processes such as lipolysis, gluconeogenesis, etc., whereas the downregulation of adipogenesis stimulates the NLRP3 inflammasome and it is responsible for adipocyte hypertrophy. In abdominal obesity, the activation of the NLRP3 inflammasome contributes to the development of a systemic chronic low-grade inflammatory state and the further deterioration of metabolic control. This is responsible for other metabolic disorders, such as metabolic-dysfunction-associated steatotic liver disease (MASLD) in humans [167] and, consequently, the onset of pathologic conditions, such as tumors. [168]. However, in obese patients subjected to a low-calorie diet and regular physical activity, a reduction in NLRP3 expression has been observed in adipose tissue [169].

The inflammation may be considered an adaptive response to overnutrition during the early phase of obesity, but as this pathologic condition progresses, the prolonged and unresolved inflammation, coupled with altered angiogenesis and adipose tissue expansion, can lead to insulin resistance, fibrosis, adipocytes dysfunction and cell death [159,169]. For this reason, developing useful strategies to prevent the inflammatory process is important as a therapeutic tool in obese patients, with particular attention also given to anti-inflammatory drugs from the plant kingdom.

## 9. PEA and Its Therapeutic Properties

**Figure 3:**
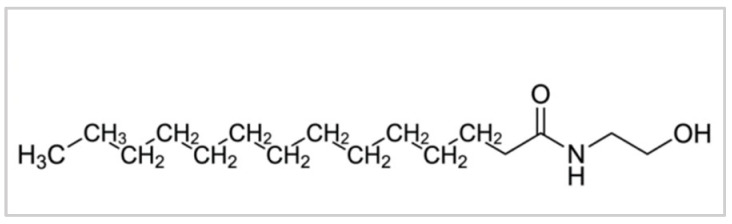
Chemical structure of Palmitoylethanolamide (PEA).

Palmitoylethanolamide (PEA), an endogenous molecule belonging to the N-acylethanolamine (NAE) family, and specifically the amide of ethanolamine and palmitic acid, is a lipid mediator [170,171]. It is considered an indirect endocannabinoid because it does not bind directly to endocannabinoid receptors but, rather, modulates their action. PEA is naturally present in small quantities in some foods, such as soy, soybeans, egg yolk, peanut meal, corn, etc. [172,173] (Figure 3). At the same time, it is a naturally occurring lipid, synthesized from membrane phospholipids in the gastrointestinal tract (although its production tends to decrease with age) and also isolated from various mammalian cells (including mast cells, macrophages, neurons, astrocytes, and microglia).

Identified in 1993 by the Nobel Prize laureate Rita Levi-Montalcini as an endogenous autacoids with an important anti-inflammatory role [174], this molecule has attracted the attention of the scientific community due to its several biological properties [175,176]. In fact, by acting on multiple molecular targets to modulate inflammatory mediators, PEA is able to exert several beneficial effects in both humans and animals (Table 2 and Table 3). More specifically, it is recognized as an endogenous lipid mediator that exerts an important effect through autacoids local injury antagonism (ALIA). It is a potent agonist of the nuclear receptor peroxisome proliferator-activated alpha (PPAR-α), considered its principal target, but, at the same time, it also acts on cannabinoid receptor G protein-coupled (GPR55 and GPR119) and it indirectly activates cannabinoid receptors (CB1 and CB2), [175,177].

Acting on the regulation of the inflammation and of the endocannabinoid system as well as on the modulation of the pain perception, PEA exhibits significant anti-inflammatory and analgesic properties and it is actually considered a promising molecule for the treatment of both chronic and acute pain in humans [178,221,222] and animals [211,212]. Several authors have investigated the mechanism responsible for its anti-inflammatory and analgesic properties, observing that PEA acts to prevent the nuclear translocation of Nuclear Factor (NF-kB) and the degradation of the inhibitory protein IkB-a, suppressing the mitogen-activated protein kinase (MAPK) and c-JunN-terminal Kinase (JNK) cascades, and reducing the inflammation in dorsal root ganglia and peripheral hyperalgesia [223]. Specifically, several studies conducted on humans and animals have documented the employment of PEA in the treatment of musculoskeletal and joint pain [179,180], fibromyalgia [181] post-operative pain [184] and osteoarthritis [178], characterized by a slow and progressive degradation of cartilage, an increased release of pro-inflammatory mediators and neuropathic pain [224]. A clinical trial demonstrated that PEA is more effective than ibuprofen in alleviating temporo-mandibular joint inflammatory pain [180]. Instead, a clinical trial on dogs with chronic osteoarthritis and persistent lameness demonstrated the effectiveness of co-ultramicronized PEA with Quercetin in reducing the severity of chronic pain and improving general animal welfare [210].

PEA is considered a novel approach for the treatment of primary headache and migraine with aura through downregulation pathways, showing a significant and time-dependent reduction in pain [185,186]. It is also useful in dysmenorrhea to reduce pain and cramping in the pelvic and lower abdominal region by preventing mast cells recruitment and activation [182,183].

Due to its anti-inflammatory properties, PEA is effective in controlling disorders characterized by neuroinflammation, a condition involving microglia and astrocytes as well as the activation of the cascade mechanism that can damage neuronal function and result in neuropathic pain, a chronic disease with major impact on quality of life, caused by a lesion or damage of the somato-sensory nervous system. It has been observed that the administration of PEA via bioavailable formulations effectively restores depleted endogenous levels, reinstaining its protective, anti-inflammatory and analgesic activities. At the same time, recent studies suggest that it could be used in modern treatment of chronic long-term inflammation, oxidative stress or mixed pain with a neuropathic component [188,189].

Another important field of application of PEA is represented by brain pathologic conditions: ultramicronized formulations of PEA have been shown to cross the blood–brain barrier, and mantein brain health in neuroinflammation and neuropathic pain, neurodegenerative disorders and neurological disturbances [225]. Specifically, ultramicronized PEA it has been shown to act on neurological illnesses linked to the development of dementia and neurodegenerative disorders, such as Alzheimer’s and Parkinson’s diseases, amyotrophic lateral sclerosis, autism spectrum disorder, etc. [191,192,193,194], by preventing striatal inflammation and inhibition of apoptosis and autophagy [226]. Furthermore, the effectiveness of its supplementation has been documented in subjects with neurological disturbance, such as anxiety, stress, cognition and attention disturbance. A study on co-ultramicronized PEA-lueolin shows its ability to increase levels of Gamma-Amino Butyric Acid (GABA) and synaptic plasticity among patients with long-COVID-19 syndrome [227]. PEA induces also inhibition of pro-inflammatory cytokines, preventing cortical spreading depression in pre-clinical studies [176]. A potential holistic use of PEA has been hypothesized as supplement against sleep disturbances due to its analgesic, anxiolytic and antidepressant properties [195,198].

The anti-inflammatory activity of PEA is also linked to the immune response: by binding to the PPAR-α receptor, it is able to modulate the release of immune cells, mainly macrophages, to enhance resistance to infection; at the same time, by inhibiting the pro-inflammatory cytokines (TNF-α, IL-1β) and the expression of adhesion molecules (ICAM-1,P-selectin) and NF-κB, it reduces the production of inflammatory signals and pain [200]. Being produced physiologically, it exerts a pro-homeostatic modulation and it does not induce immune suppression. The ability of PEA to reduce inflammation and immune responses through immunomodulation, such as its mast-cell-modulating properties, has been documented in several studies [199,228,229]. Through an immunemodulation involving macrophages, PEA is also able to exert a protective action against microbial infections, increasing the not-specific (innate) resistance to bacteria systemic infections [230]; it was shown to be safe and effective in flu, common cold and respiratory infections [200,201]. In addition, the inhibitory effect of PEA on the immunologically induced histamine, prostaglandin-2 and TNF-α release has been observed *in vitro* on canine mast cells freshly isolated from skin [214] and on the cutaneous allergic inflammatory response *in vivo* in hypersensitive beagle dogs [215].

PEA has shown anti-allergic effects in the presence of inflammatory cell infiltration in allergic rhinitis [203], dermatitis [202], and asthma [204], through direct activation of PPAR-α and GPR55 receptors and the indirect activation of cannabinoid receptors (CB1 and CB2). Recently, the possible use of PEA in nickel allergy has been evaluated as a potential therapeutic strategy to protect barrier function and mitigate the cutaneous and gastrointestinal damage consequent to this allergic condition through the modulation of mast cell activity [205]. *In vitro* studies on canine skin mast cells showed that PEA induces a significant and dose-dependent inhibition of PGD2, TNF-α and histamine release [214]. Clinical studies also documented the effectiveness of PEA in reducing skin lesions in dogs with atopic dermatitis and moderate pruritus [216], and in cats with allergic dermatitis [217].

In addition, PEA plays an important role in the regulation of gut microbiota [206,207], acting on acute and chronic inflammation of the gastrointestinal tract caused by dysbiosis or vitamin D deficiency, which are responsible for damage to the epithelial barrier. It is also able to increase the presence of *Enterobacteriaceae* spp. and other common bacteria, as documented in animals [206]. Furthermore, the potential therapeutic use of PEA as a dietary supplement has been evaluated to counteract gastrointestinal disorders and to compensate for altered gut homeostasis through its exogenous intake [231]. The effectiveness of PEA against enteric inflammation associated with bowel motor dysfunctions in Alzheimer’s disease has been demonstrated *in vitro* in an experimental model of senescence-accelerated mice [232]. The enteroprotective effect of PEA is probably due to the activation of PPAR-α and CB2 receptors in both humans [231] and animals [219]. In a study carried out on dogs with chronic diarrhea supplemented with micronized PEA, a reduction in Canine Inflammatory Bowel Disease Activity Index (CIBDAI) score was observed [220]. Similarly, a supplement containing ultramicronized PEA has been shown to exert beneficial effects on the gut health of weaning puppies, decreasing two markers of intestinal damage (i.e. zolnulin and calprotectin) [233]. Another study on animals with experimentally induced colitis demonstrated the role of PEA in mitigating this pathological condition [209]. In addition, for its anti-inflammatory, analgesic, antimicrobial, and immunomodulatory effects, PEA can improve pathologic conditions of gastrointestinal tract, particularly irritable bowel syndrome and inflammatory bowel diseases, ulcerative colitis, and Crohn’s disease [209,231].

Finally, in literature there are also *in vitro* study on PEA antioxidant activity, which need to be indeept, to better understand its true rule in oxidative stress [234,235].

## 10. PEA in the Treatment of Obesity

Recently, due to the high incidence of obesity and its comorbidities, natural products obtained from plants are considered valid tools to counteract this pathologic condition. Several compounds present in fruits, vegetables, and edible plants, such as anthocyanins, epigallocatechins, polyphenols, saponins, curcumin and PEA, have shown anti-obesity activity both *in vivo* and *in vitro* [236,237,238,239,240,241,242,243,244]. These active products are able to decrease fat accumulation through different mechanisms [239], such as the inhibition of adipocytes differentiation, adipogenesis, the reduction in triacylglycerol levels in high-fat diets, the enhancement of lipolysis, or the reduction in lipogenesis.

PEA, which is also an endogenous lipidic mediator secreted by adipose tissue [245], is an object of interest for its role in obesity because, together with other compounds of the NAEs family, it is involved in the regulation of feeding, energy and fat metabolism [246]. For example, Anandamide (AEA) stimulates energy intake and storage, N-oleoylethanolamide (OEA) mediates anorectic signals and lipidoxidation, while PEA counters inflammation linked to obesity. It is known that circulating ECs and their congener NAEs are metabolically connected to diet (appetite, regulation of energy homeostasis, inflammation, obesity and dysmetabolism), nutrition, metabolic and hormonal axes [247,248,249,250] and, consequently, to metabolic disorders [251]. In particular, plasmatic EC and NAE concentrations have been investigated as potential biomarkers of WAT distribution in obese subjects [252,253,254]. Some authors have studied levels of PEA and other NAEs in human plasma [250] and saliva [254] to evaluate their association with obesity.

The correlation between PEA and obesity is not entirely clear, but it has been observed that its administration determines health benefits through the regulation of gluco-lipid metabolism and other pathways. In particular, PEA reduces circulating inflammatory markers like TNF-α, IL-6 and CRP in the treatment of obesity-related inflammatory diseases [255]. It was documented that the intestinal PEA levels increase in the postprandial phase and are able to regulate food intake in rats [171], while their decrease is correlated to dietary fat consumption [256,257].

PEA, being a potent agonist of the transcription factor PPAR-α in hepatocytes, is able to regulate several pathways involved in obesity. Specifically, it controls cellular energy requirements, lipid and glucose metabolism [171,258,259], regulates food intake and release of gut peptides, and indirectly influences the expression of genes involved in lipid metabolism, distribution and storage of free fatty acids (FFAs) [260]. Through PPARα activation, PEA also mediates the interaction between the liver and adipose tissue in regulating whole-body energy metabolism, which is a crucial step in adipose tissue plasticity, as it is involved in the leptin-mediated lipolytic effect [261,262]. In addition, PPAR-α modulates the release of fatty acids, the main fuel for thermogenic activity, and stimulates gene pathways involved in the production of BAT [263,264]. Considering that PPAR-α is involved in whole-body homeostasis and lipid and glucose metabolism, and its activation can be considered an important target for MASLD therapy, the regulatory role of PEA in the transcription of PPAR-α in hepatocytes could explain its use in the treatment of obesity.

Some authors reported that supplementation of ultramicronized-PEA (30 mg/kg/day orally for 7 weeks) in high-fat-diet-induced obese mice contributes to the conversion of WAT tissues through PPAR-α activation, leading to the alteration of energy homeostasis, prevention of fat accumulation and modulation of adipocytes differentiation. In particular, in this study, PEA treatment significantly restored the mRNA levels of regulator genes associated with BAT-thermogenesis (*Ucp1*, *Ppargc1a*, *Prdm16* and *Cox8b*), and immunohistochemical evaluation revealed an increased density of parenchymal noradrenergic nerves in the BAT of treated obese mice; in addition, the light microscopy imaging showed a reduction in cytokine transcription (IL-6 and TNF-α) and an increase in adiponectin levels in obese mice after PEA treatment [265].

In a study by Hoareau et al. [245] regarding the effects of PEA on TNF-α secretion in human adipocytes, its ability to inhibit LPS-induced secretion of TNF-α was documented, highlighting the potential role of PEA in the prevention of obesity-associated insulin resistance. Hoareau and Roche [21], instead, reported that the treatment of human adipocytes with leptin is responsible for a downregulation of PEA synthesis, correlated to the expression of the PPARα.

Although PEA is not directly involved in appetite regulation and weight gain, some studies have also documented its effect on food intake and body weight as well as its interaction with hypothalamic leptin signaling [266]. In particular, the preventive action of PEA on leptin resistance has been observed in nonobesogenic conditions and confirmed in vitro using a model of SH-SY5Y neuroblastoma cell line to study leptin signaling [267].

The mechanism of action by which PEA regulates feeding behavior is not fully understood. However, a study by Mattace Raso et al. [171] carried out on an ovariectomy-induced rats model treated with PEA (30 mg/kg subcutaneously for 5 weeks) showed anorectic and fat-loss effects, with a recovery of the impairment of leptin, increase in glucose tolerance, and decrease in food intake, body weight, and fat mass.

Great attention is also focused on the effect of ultramicronized PEA on the regulation of gut microbiota, particularly on the population of *Enterobacteriaceae* spp. and other common bacteria [206], to avoid inflammatory stimuli and prevent high-fat-diet-induced gut dysfunction [268].

Some authors carried out *in vitro* studies on Caco-2 cell lines, *ex vivo* studies on human mucosa, and a randomized, double-blind, controlled trial in humans treated with aspirin, documenting that PEA can prevent intestinal damage and inflammation involving PPARα [207].

Recently, the ability of ultramicronized PEA to avoid gut dysfunction in obese mice was observed by reducing intestinal immune cell recruitment and inflammatory response as well as rebalancing the composition of the gut microbiota [268]. In particular, in an experimental model in mice fed a high-fat diet (HFD), the supplementation with ultramicronized PEA (30 mg/kg/day orally for 7 weeks) showed beneficial effects on intestinal inflammation and microbiota alterations by modulating altered tryptophan metabolism in the colon, rebalancing serotonin turnover and promoting/producing bacteria, such as *Bifidobacterium*, *Oscillospiraceae* and *Turicibacter sanguinis*.

Finally, the administration of PEA could also influence the hepatic metabolism, altering the fatty acids, glucose, and aminoacid levels, and improve the hepatic health [244], preventing fibrosis, hepato-cellular carcinoma, and other liver diseases. 

**Figure 4:**
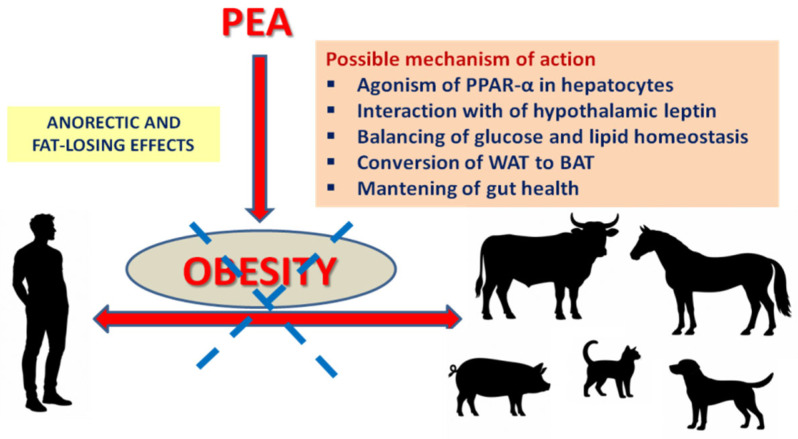
Possible mechanism of action of PEA in counteracting human and animal obesity.

Figure 4 summarizes all the possible mechanism of action of PEA in human and animal obesity.

### 10.1. PEA in Human Obesity

An interesting approach in the management of human obesity is the use of food supplements of natural origin, such as vitamin D, Coenzyme Q10, turmeric, quercetin, ginger, probiotics, PEA, etc. [269,270,271,272]. Among these, PEA is able to act on inflammation associated with obesity, reducing circulating inflammatory markers like TNF-α, IL-6 and CRP.

As previously reported, ECs and NAEs are metabolically connected to diet, and their plasmatic concentrations are considered biomarkers of WAT distribution in relation to blood cholesterol and insulin resistance in obesity. The exact role of PEA in human overweight and obesity remains unclear and it is currently the object of study, although research carried out in adipocytes and human cell cultures has provided interesting results [21,245].

Few investigations, however, have been conducted in human patients with overweight and/or obesity. A study carried out in a Brazilian population of complex interethnic admixture with a wide BMI range documented the genetic variants of the EC components in the susceptibility to obesity and related metabolic disorders, identifying an endocannabinoid-related obesity phenotype [247]. Another cohort study on PEA in obese patients comprising 184 females (premenopausal and menopausal) and 144 males stratified into normal weight (BMI: 18.5–24.9 kg/m^2^), overweight (BMI: 25.0–29.9 kg/m^2^), and obese (BMI > 30.0 kg/m^2^) showed a not-defined association of plasmatic NAEs with human obesity and cardio-metabolic risk [250]. These data contrast with findings in previous studies, possibly caused by heterogeneous population selected, experimental designs or unstandardized NAE measurements [273].

In a double-blind study, some authors evaluated the effects of PEA on the gut microbiome and biochemistry in an overweight adult population and documented that its supplementation (350 mg orally once daily for 12 weeks) may influence specific microbial species and metabolic pathways as well as reduce serum triglycerides and IL-2, underlying its potential therapeutic use in pathologic conditions correlated to overweight, such as metabolic inflammation and obesity [274].

Other authors hypothesized the use of PEA in postmenopausal metabolic syndrome and its associated comorbidities (hypertension, abdominal obesity, insulin resistance, dyslipidemia, etc.) [275], through evidence from *in vitro* studies and in animal experimental models. Due to the lack of clinical studies, and although data present in the literature suggest a possible use of PEA in the range of 300-1200 mg daily, today, its optimal dosage remains unclear due to the low bioavailability and poor water solubility of most formulations [175,275]. However, considering its important role in regulating lipid metabolism, inflammation, and energy homeostasis, PEA may be useful to counteract postmenopausal metabolic syndrome due to a decline in estrogen, acting through several pathways: adipose tissue remodeling, improvement of lipid profile and hepatic function, weight management, appetite regulation, enhancement of insulin sensitivity, etc. [275]. In particular, PEA could act against the reduced metabolic rate associated with hormonal changes, influencing the adipose tissue to produce adipokines, involved in the development of metabolic syndrome [276]; at the same time, it can also participate in cholesterol regulation and metabolism, lowering LDL levels and improving lipid profiles [277]. Given its poor side effects, the use of PEA as a supplement in the management of this syndrome could be useful in long-term therapy for women with a complex symptomatology. In addition, considering also its several biological properties and its ability to reduce neuropathic pain, inflammation and oxidative stress, PEA could enhance the frequency and/or intensity of physical activity in women with metabolic syndrome, improving muscle function and recovery after exercise.

A recent randomized, crossover, double-blind, place-bo-controlled study investigated the effects of a co-micronized PEA-rutin combined with hydroxytyrosol, administered together with a tailored calorie-controlled Mediterranean diet, in patients with metabolic syndrome. The supplemented patients showed a significant reduction in body weight, body mass index, fat mass, and inflammation biomarkers, along with increases in fat-free mass, phase angle, and body cell mass compared with placebo group. These preliminary findings suggest a supportive role for co-micronized PEA–rutin and hydroxytyrosol in metabolic syndrome [278].

Currently, PEA is not yet widely employed as a coadjuvant in the pharmacological therapy of overweight and obesity in humans, probably due to the limited knowledge on the link between PEA and these pathologies. For this reason, clinical studies should be carried out using novel formulation [279] and standardized protocols, taking into consideration the promising results and the clinical applications of PEA in veterinary medicine.

### 10.2. Use of PEA in the Treatment of Animal Obesity

Considering the effectiveness of biologically active molecules from the plant kingdom and the most recent approaches to “green pharmacology”, PEA is considered a valid tool in the treatment of animal obesity [280] and is effective in obesity-induced meta-inflammation [281].

Few data have been reported in the literature on the effects of PEA in obese animals, and its mechanism is not exactly clear, although it appears to be indirectly implicated in appetite regulation and weight gain. Some authors have documented the metabolic effects of PEA in animal models of obesity, showing that it reduced food intake and fat mass. It was also shown to modulate hypothalamic neuropeptide transcription and to decrease the inflammation in adipose tissue of ovariectomized rats [170]. At the same time, PEA rebalances the glucose and lipid homeostasis, avoiding consequent hepatic metabolic alterations, in mice fed a high-fat diet [265]. A study carried out on Sprague-Dawley rats fed a hypercaloric cafeteriadiet (5.3 kcal/g) showed that PEA (injectedi.p.at a dose of 10 mg/kg b.w. for 15 days) is able to improve the health status in this animal model of diet-induced obesity [246]: it decreases weight, liver steatosis, inflammation, and dyslipidemia by acting on PPAR-α through a mechanism of action similar to that of OEA, well known for the control of appetite, lipid metabolism, and inflammation [282].

Considering the increased incidence of obesity in veterinary medicine, and the importance of diet in the management of animal obesity [283], it is possible to admit the use of PEA in dietary supplements for pets, associated with a balanced diet and calorie restriction [284]. In particular, some authors described the synergistic effects of the co-micronization of PEA with antioxidants, such as Rutin and Hydroxytyrosol (PEA 10 mg/kg/day; Rutin 2 mg/kg/day and HT 0.5 mg/kg/day orally for 12–19 weeks), administered to obese mice fed an HFD, against hepatic damage and metabolic alterations [260]. Other authors reported the effectiveness of omega-3 supplementation in peripartum Holstein dairycows (300 g/d per cow with encapsulated fat providingα-linolenic acid (ALA) at 56.1 g/d, and PP at 700g/d per cow providing 131.0 g/d ALA) on components of the endocannabinoid system to improve the metabolic and inflammatory responses in adipose tissue and to reduce food intake by 8.1% [285].

Another important aspect in the use of PEA in animal obesity is that, as a bioactive endogenous molecule of the EC system acting as prohomeostatic compound for inflammation, metabolism and energetic homeostasis, its administration could also be beneficial for improving other disorders consequent to overweight and obesity, particularly joint and cartilage pain, bone degeneration, arthritis, and musculoskeletal alterations.

Recently, a new formulation has been approved for use as complementary feed for overweight and obese dogs andcats. This formulation, consisting of Olaliamid^®^, an olive-oil-derivate NAEs mixture (OEA, PEA and LEA in the same ratio as the fatty acids present in olive oil) and vitamin E, is able to reduce the risk of developing overweight and obesity related disorders, reduce systemic inflammation, and neutralize free radicals, improving oxidative balance and enhancing lipid and glucose metabolism. Consequently, it protects liver function, supports cardiovascular health (also in the absence of weight loss), and maintains the adipose tissue homeostasis in dogs and cats, in association with a safe and well-regulated diet and physical exercise.

A study by Melini et al. [286] documented the effectiveness of Olaliamid^®^ on insulin resistance and adipocyte reprogramming in HFD-fed mice. Animals treated with Olaliamid^®^ (10 mg/kg/day orally by gavage for 12–19 weeks, fed with HFD for 7 weeks) showed a significant reduction in obesity and improvement in glucose tolerance, restoring biochemical, hormonal, and inflammatory parameters to baseline levels. This treatment was able to increase the transcription of thermogenesis BAT-associated genes and to reduce the RNAs levels of IL-1β and GDF15 in brown adipocytes of obese mice, limiting systemic and tissue inflammation correlated to obesity [286]. Furthermore, in a double-blind, randomized, placebo-controlled study by Piantedosi et al. [287], the effect of Olaliamid^®^ was evaluated in obese dogs. This supplement, administered orally by the owners in a liquid form (0.7 mL/5 kg b.w. once daily for three months), prevented increases in leptin and improved ALT, urea and bilirubin serum levels demonstrating its safe use and beneficial effects against obesity-induced low-grade chronic inflammation (meta-inflammation) and oxidative stress, also improving liver and heart health.

In general, studies with larger sample sizes of animals are needed to better elucidate the real mechanisms of PEA and NAEs as coadjuvants in the treatment of obesity.

## 11. Limitations

Despite promising anti-obesity properties, the use of PEA in the management of overweight and obesity remains limited. Novertheless, the preliminary results on PEA—either co-micronized with rutin or as part of the Olaliamid® mixture—show promise in both in human and veterinary medicine The main limitation is the scarcity of clinical trials on humans and animals, and the small sample sizes of the studies conducted so far. For this reason, most of the articles selected for this review are related to studies on cell cultures and animal experimental models used to explain the mechanism of obesity, the link between obesity and inflammation, and the anti-obesity properties of PEA. Certainly, more in-depth knowledge of the effects of PEA on inflammation correlated with obesity could contribute to its use, as a dietary supplement or coadjutant to pharmacological treatment with drugs actually available to counteract overweight and obesity. However, an important limitation in the use of PEA is represented by its pharmacokinetic features. Specifically, unprocessed PEA exhibits poor bioavailability upon oral delivery, insolubility in the gastrointestinal tract, and a relatively short half-life; furthermore, it is rapidly metabolized and excreted once absorbed [225]. In this context, the research on PEA is currently focused on the development of new formulations, such as micro- and nano-systems, to increase its absorption and bioavailability after oral delivery. Briskey et al. [288] demonstrated, in a clinical trial carried out on healthy humans, an increased absorption of PEA formulation using a novel dispersion technology system. Recently, Mehkri et al. [289] studied the pharmacokinetics of micronized, water-dispersible and standard formulations of PEA after single oral administration in male Sprague-Dawley rats, documenting a higher bioavailability of water-dispersible PEA, followed by micronized system (PEA-6 μm and PEA-10 dμm) (with greater total AUC and Cmax), compared to traditional formulation. Jose Louis et al. [290] studied the pharmacokinetics of a reversible hybrid hydrogel (N’SERH) formulation in a randomized, single-dose, double-blinded trial on healthy human volunteers, showing an enhancement in PEA bioavailability. The study of Khan et al. [291], instead, proposed a new phospholipid-based PEA formulation to increase solubility, distribution and clinical effects in chronic treatment of pain. Therefore, considering the results obtained in these *in vivo* and *in vitro* studies, the new technologies represent a valid strategy to improve the pharmacokinetic profile of PEA and, consequently, its therapeutic use, in both human and veterinary medicine. Particularly, micronization offers an advantage over hydrodispersions and lipodispersions by enhancing bioavailability through the physical reduction of particle dimensions, thereby overcoming the limitations associated with poor oral drug delivery. Consequently, ultramicronized formulations show high bioavailability and efficacy, demonstrated to successfully penetrate the CNS [292]. 

However, further clinical studies should be carried out in humans and different animal species (considering all the species-specific variables), to support the clinical use of PEA in the treatment of overweight, obesity and related comorbidities and in multidrug therapies.

## 12. Considerations and Perspectives

The fight against obesity represents an issue of global interest because, despite current knowledge and efforts made through informational campaigns, this disease remains significantly widespread. The most recent increase in human obesity incidence is accompanied by diffusion of this pathology also in the veterinary field, particularly in pets; therefore, animals’ health status frequently reflects that of their owners. For this reason, it is important to carry out communication among the food industry, dietitians, clinics, schools and institutions to promote healthy dietary habits through human and animal educational programs. In this context, a useful approach to counteract overweight and obesity is certainly to promote healthy eating habits and valid dietary models, such as the Mediterranean diet. The Mediterranean model, in fact, aims to reduce the caloric intake and the consumption of saturated fatty acids, replacing these last with polyunsaturated fatty acids or monounsaturated fatty acids and preferring a relatively high amount of carbohydrates. This diet, proposed by Keys in the last century, continues to be of great actuality because it does not represent only a food model but, rather, the lifestyle of Mediterranean populations. It provides a correct and balanced intake of essential nutrients, associated with regular physical activity (historically represented in this model by agricultural field work), healthy outdoor living and respect for nature [293].

Several studies have provided scientific evidence of the effects of the Mediterranean diet on weight loss and a reduction in central adiposity. For instance, the results obtained in the study by Beunza et al. [294] showed that the subjects with the lowest adherence to Mediterranean diet had the highest initial weight gain; conversely, those with the highest adherence to this dietary pattern had good weight maintenance. In addition, the high assumption of plant-derived food (vegetables, fruits, whole grains, and nuts) proposed in this model presents several health benefits, particularly against obesity and related pathologies, such as inflammation, thrombosis, insulin sensitivity, hypertension, cardiovascular diseases, endothelial dysfunction and gut microbiota alterations [295]. As is known, the success of the Mediterranean diet is attributable to the richness of the biologically active substances present in plant-derived food (anthocyanins, epigallocatechins, saponins, curcumin, PEA, etc.), known for their various healthy properties, including anti-obesity effects [236,237,238,239,240,241,242,243,244]. Relating to PEA, a study of Tagliamonte et al. [296] underlined its link with the Mediterranean diet in relation to health status, documenting that this dietary model affected the plasma concentrations of ECs and NAEs, ameliorating insulin sensitivity and reducing systemic inflammation.

As discussed in this review, the data available in the literature on humans and animals demonstrate that PEA is a promising tool in the treatment of overweight and obesity, particularly as a food supplement currently used in veterinary medicine. Certainly, the adherence of the population to a balanced diet, as proposed by the Mediterranean model, and the choice of safer, unprocessed and so-called “km 0” foods, is a valid strategy in weight control. These safe and balanced food habits, together with regular physical activity and a lifestyle in balance with nature, following the most recent *One-health* vision, admit to owners to protect their health and, at the same time, that of their animals, considering that the onset of obesity could be recognized as a symbiotic relationship between human and animal.

In conclusion, the use of PEA as a dietary supplement for its anorectic and fat-losing properties could be considered a valid strategy to counteract overweight and obesity in humans and animals. Its consumption could be also combined with probiotics to support gut microbiome health, to prevent gut disorders and metabolic syndrome, and to avoid the onset of other consequent comorbidities.

## Figures and Tables

**Table 1:** Drugs used in the treatment of overweight and obesity in humans.

Anti-Obesity Drugs	Class	Effects	References
PHENTERMINE	Sympathomimetic	Appetite suppression through norepinephrine, dopamine and serotonin release in the CNS.	[103]
DIETHYLPROPION	Sympathomimetic	Appetite suppression through norepinephrine, dopamine and serotonin release in the CNS.	[103]
TOPIRAMATE	Carbonic anhydrase inhibitor	Reduction in appetite and binge-eating behaviors.	[104]
BUPROPION	Antidepressant	Anorectic mechanism mediated by the inhibition of dopamine and reuptake of norepinephrine.	[105]
NALTREXONE	Antagonist of opioid receptor	Appetite suppression through disruption of β-endorphin-mediated.	[106]
SETMELANOTIDE	Agonist of melanocortin-4-receptor	Reduction in appetite and regulation of energy expenditure via action on several hypothalamic areas.	[107]
METRELEPTIN	Analog of leptin	Mimic action of leptin and improvement of Metabolic parameters.	[108]
ORLISTAT	Inhibitors of lipase	Prevention of fat intestinal absorption and of caloric intake without affecting appetite.	[109]
CETLISTAT	Inhibitors of lipase	Prevention of fat intestinal absorption and of caloric intake without affecting appetite.	[110]
LIRAGLUTIDE	Agonist of glucagon like peptide receptor (GLP-1)	Reduction in blood glucose levels, increase in satiety and decrease in appetite, promotion of slow gastric emptying and reduction in body weight.	[111]
SEMAGLUTIDE	Agonist of glucagon like peptide receptor (GLP-1)	Reduction in blood glucose levels, increase in satiety and decrease in appetite, promotion of slow gastric emptying and reduction in body weight.	[112]
TIRZEPATIDE	Agonist of GLP-1 and glucose-stimulated insulin-tropic peptide receptor	Stimulation of pancreatic insulin release, reduction in hyperglycemia and increase in adiponectin levels.	[113]
GELESIS	Hydrogel particles, with similar effects of raw vegetables	Distension of stomach and small intestine during the meal, promotion of fullness and reduction in appetite.	[114]

**Table 2:** PEA use in different human diseases.

USE	HUMAN DISEASES
**ANTI-INFLAMMATORY** **AND ANALGESIC EFFECTS**	Osteoarthritis [178], skeletal muscle hypertrophy [179], temporo-mandibular joint pain [180], fibromyalgia [181], dysmenorrhea [182], endometriotic pain [183], post-operative pain [184], headache and migraine [185,186].
**NEUROPHATIC** **PAIN MODULATION,** **NEURODEGENERATIVE** **DISORDERS AND** **NEUROLOGICAL** **DISTRUBANCES**	Chronic pain [187,188], peripheral neuropathy [189], antinociceptive effect in neuropathic pain [190], Alzheimer’s disease [191], Parkinson disease [192], multiple sclerosis [193], autism spectrum disorder [194], anxiety [195], depression [196,197], sleep and recovery [198].
**IMMUNO-RESPONSE**	Immunomodulation [199], flu and common cold [200], respiratory infection [201].
**ANTI-ALLERGICEFFECTS**	Contact allergic dermatitis [202], allergic rhinitis [203], asthma [204], nickel allergy [205].
**GASTROINTESTINAL** **DISTRURBANCE**	Gut microbiota regulation [206,207], irritable bowel syndrome and inflammatory bowel diseases [208], ulcerative colitis [209].

**Table 3:** PEA use in different animal diseases.

USE	ANIMAL DISEASES
**ANTI-INFLAMMATORY** **ANDANALGESIC EFFECTS**	Chronic osteoarthritis and lameness in dogs [210]. Inflammation and pain in companion animals [211].
**NEUROPHATIC** **PAIN MODULATION**	Chronic pain in dogs [212] and cats [213].
**IMMUNOMODULATION**	Immunologically response in canine skin mast cells [214].
**ANTI-ALLERGIC EFFECTS**	Cutaneous allergic inflammatory response in beagle dogs [215], atopic dermatitis and moderate pruritus on skin lesions in dogs [216] and in cats [217]
**GASTROINTESTINAL** **DISTURBANCES**	Gut microbiota regulation [218], canine enteropathies [219], canine chronic diarrhea [220].

## Data Availability

No new data were analyzed or created during this study. Data sharing is not applicable.

## Abbreviations

The following abbreviations are used in this manuscript:

FDA	Food and Drugs Admnistration
PEA	Palmitoylethanolamine
WHO	World Health Organization
WAT	White Adipose Tissue
BAT	Brown Adipose Tissue
BMI	Body Mass Index
TNF-α	Tunor necrosis factor-α
IL-6	Interleukine 6
IL-8	Interleukine 8
AGEs	Advanced Glycated End-products
ROS	Reactive Oxygen Species
BCS	Body Condition Score
CNS	Cresty Neck Scores
AOM	Anti-Obesity Medications
LEPR	Leptin Receptor
CRP	C-Reactive Protein
NLRP3	Nod-Like Receptor Protein 3 inflammasome
MASLD	Metabolic Dysfunction-Associated Steatotic Liver Disease
NAEs	N-acylethanolamine
ECs	Endo-Cannabinoids
PPAR-α	Peroxisome Proliferator-Activated Receptor-α
